# Three-Dimensional (3D) Printing in the Case of a Concurrent Polyp and an Ectopic Tooth

**DOI:** 10.7759/cureus.53681

**Published:** 2024-02-06

**Authors:** Nesha Rajendram, Liang Chye Goh

**Affiliations:** 1 Otorhinolaryngology, Faculty of Medicine Universiti Teknologi MARA, Selangor, MYS; 2 Otorhinolaryngology, Faculty of Medicine University of Malaya, Kuala Lumpur, MYS

**Keywords:** transanal endoscopy, paranasal sinus diseases, ‎3d printing, ectopic tooth, polyps

## Abstract

The removal of an ectopic molar tooth at the pterygomaxillary junction may be challenging. This paper presents the use of three-dimensional (3D) printing of the paranasal sinus for careful planning in a way that reduces the risk and makes surgical procedures more effective. A 26-year-old gentleman presented to the ENT department with a left antrochoanal polyp and an incidental ectopic tooth at the pterygomaxillary junction. Pre-operative 3D reconstruction of the maxillary cavity and subsequent 3D printing were used to guide the surgery and counsel the patient on potential outcomes. Left anterior functional endoscopic sinus surgery was subsequently done, and the antrochoanal polyp was completely removed. The preoperative computed tomography scan allowed for the production of the printed model to the exact size and dimensions of the ectopic molar tooth to facilitate the planning of the surgery and to aid in consenting the patient for the treatment.

## Introduction

Ectopic teeth are those that do not fit in their normal location in the oral arch. Developmental defects, genetic variables, infections, or iatrogenic reasons (such as trauma or surgical treatment) are only a few of the causes that might lead to this. They can result in local sinonasal symptoms and diseases when they erupt into the maxillary sinuses. When surgical removal is an option, it should be used to address this pathological disease in the maxillary sinus because, if left untreated, it has the propensity to develop into a cyst or tumour and obliterate the nasal cavity [1]. The pterygomaxillary fissure, which contains numerous significant neurovascular pathways, and the complicated and convoluted anatomy of the maxillary bone make the surgery much riskier and more difficult. This is when thoughtful planning helps to reduce this risk and improve the efficacy of surgery. Using contemporary imaging and modelling techniques, this risk can be reduced, and the results can be enhanced. Although 3D printing in medicine is still a relatively new technology, it has already become widely used, especially for anatomical "study-modelling," which involves using models for both surgical planning and the actualization of anatomy [2]. In this case report, we show how accurate preoperative surgical planning could be carried out to give the patient the best results through the reproduction of ectopic tooth morphology and maxillary architecture with the use of adjunctive 3D printing.

## Case presentation

**Figure 1:**
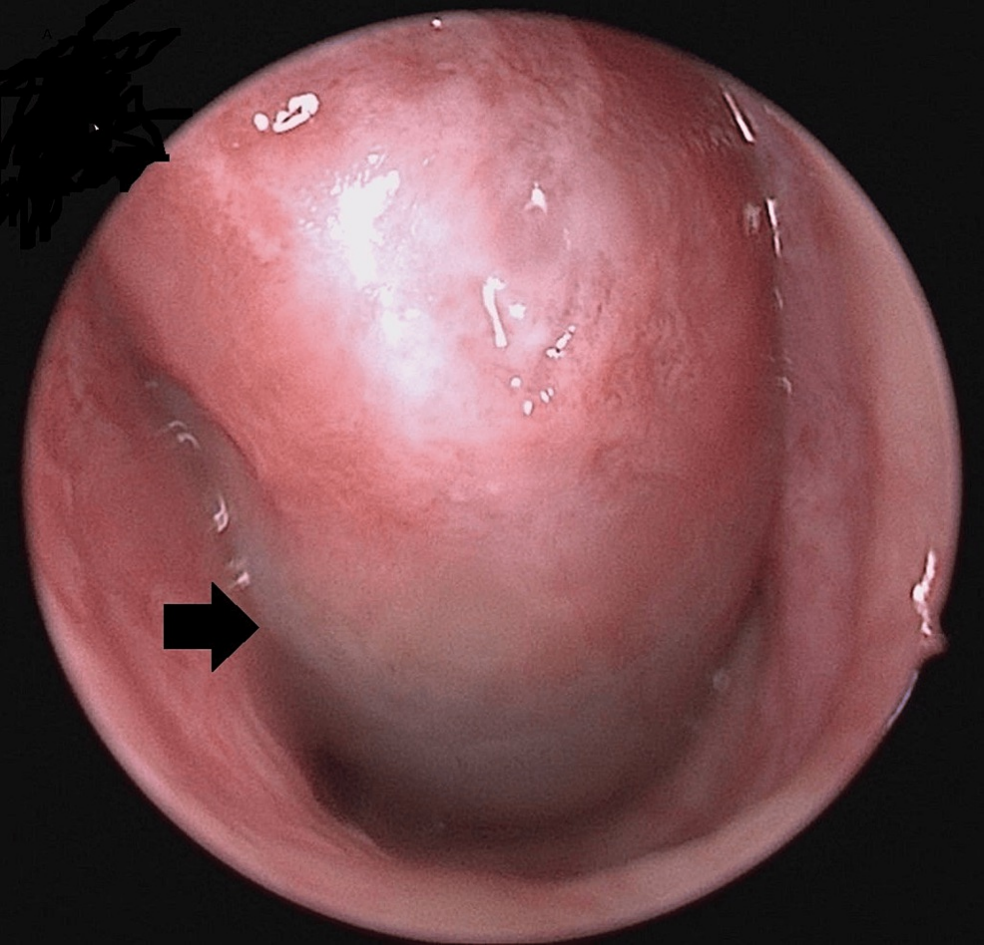
Nasal endoscopic view The image represents the left antrochoanal polyp (black arrow).

A 26-year-old man with an underlying repaired atrioventricular septal defect presented to the otorhinolaryngology (ORL) clinic with a four-year history of left nasal obstruction, cacosmia, and intermittent epistaxis. Initially, he was treated for chronic rhinosinusitis, but his symptoms have worsened over the past two years. The clinical examination revealed no facial sensitivity or facial asymmetry. The examinations of the facial nerve, orbit, and mouth were unremarkable. Initial rigid nasoendoscopy of the left nasal cavity revealed an antrochoanal polyp completely obstructing the nasal cavity (Figure 1).

**Figure 2:**
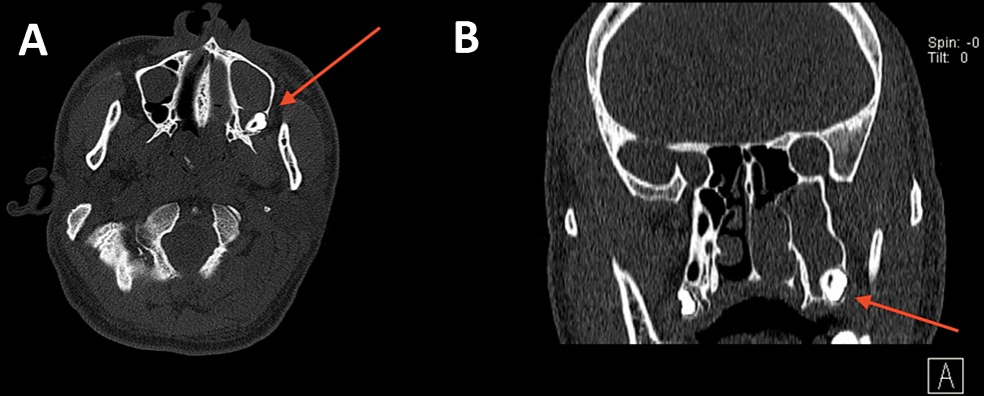
CT image of the paranasal sinus (2A) shows an axial view of the ectopic tooth (arrow) at the left postero-inferior maxillary sinus and (2B) shows a coronal view of the ectopic tooth (arrow) at the left postero-inferior maxillary sinus.

It was determined that the nasal polyp originated from an ectopic molar tooth in the postero-inferior region of the maxillary cavity. The right nasal cavity was clinically normal. CT scans of the paranasal sinuses reveal a non-contrasting hypodense lesion extending from the left maxillary cavity to the nasal cavity with an underlying molar ectopic tooth at the postero-inferior region (Figures 2A, 2B).

**Figure 3:**
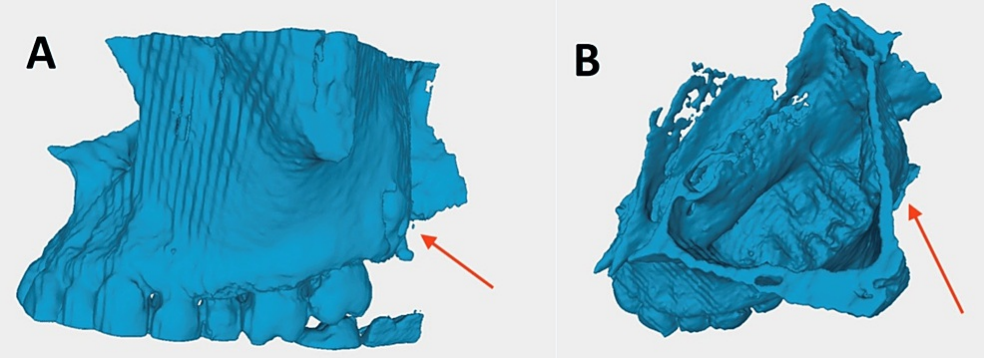
Stereolithographic images (3A) shows the lateral view of the STL (stereolithographic) image of the 3D reconstructed model with the ectopic tooth (red arrow) embedded adjacent to the pterygomaxillary junction and (3B) shows the superior view of the STL image of the 3D reconstructed model with the ectopic tooth (red arrow) embedded adjacent to the pterygomaxillary junction.

**Figure 4:**
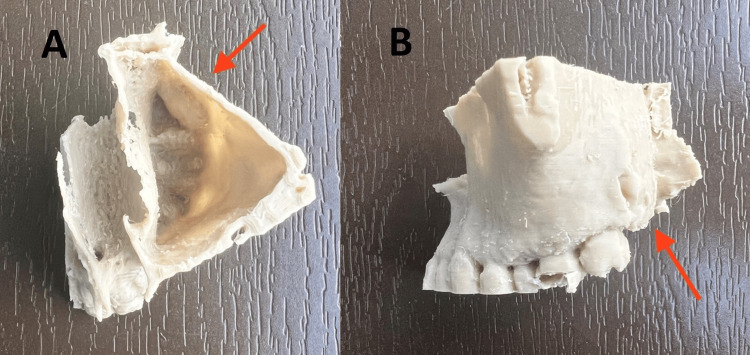
3D printed model using PLA material (4A) shows the superior view of the three-dimensional (3D) printed model using polylactic acid (PLA) material with the location of the ectopic tooth in relation to antrochoanal polyp (red arrow) and (4B) shows the lateral view of the 3D printed model using PLA material with the location of the ectopic tooth in relation to antrochoanal polyp (red arrow).

A biopsy of the left nasal polyp revealed inflammatory polyps with atypia of the stroma. The construction of a 3D model was a part of the specialised research conducted preoperatively to evaluate the prospective surgical options (Figures 3, 4).

**Figure 5:**
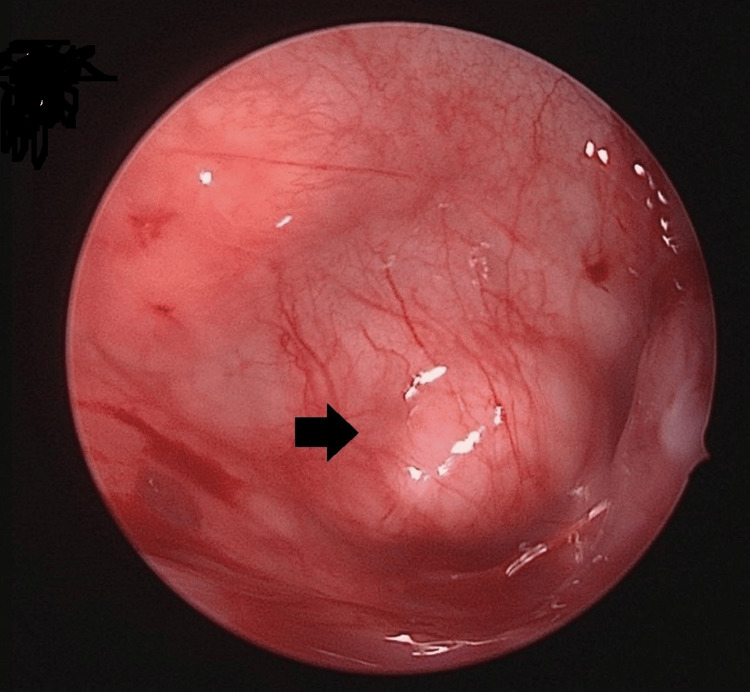
Intraoperative endoscopic image Intraoperative endoscopic image of left intramaxillary ectopic tooth with its overlying mucosa (black arrow).

He underwent left anterior functional endoscopic sinus surgery whereby the maxillary natural ostium was widened and the uncinate process was removed. The antrochoanal polyp was completely removed via the trans-nasal route. Intraoperatively, a left nasal polyp was observed to occupy the left nasal cavity until the posterior choana, with its epicentre in the left maxillary cavity. There was a marsupialized cyst at the maxillary antrum from which clear serous fluid was extracted. In addition, there was a root of the left upper third molar at the floor of the left maxillary sinus with intact mucosa overlying the tooth (Figure 5).

Histopathological analysis revealed the presence of inflammatory nasal polyps. An unerupted ectopic molar complicated by an anthrochoanal polyp of the left maxillary sinus was the diagnosis. Due to the close proximity of the ectopic tooth to the pterygomaxillary fissure and the patient's resolution of symptoms following surgery, he consented to close monitoring and routine nasal douching to detect the recurrence of nasal polyps. A dental consultation was also provided for a second opinion on the treatment of ectopic teeth.

## Discussion

The process of developing teeth is multi-step, and interactions between the oral epithelium and mesenchymal tissue occur throughout. Ectopic development can be caused by any aberrant tissue contact while the process is happening [3]. Even though ectopic tooth eruptions are frequently observed in the dentate region, they are still extremely uncommon in the nondentate area [4]. The mandibular condyle, coronoid axis, palate, maxillary sinuses, and nasal cavity are some areas of the jawbones where the ectopic tooth can be discovered and are relatively remote from the arch. Due to the rarity of teeth being found within the sinus, they are often found during routine clinical or radiographic examinations [5]. Rarely, ectopic teeth can cause temporomandibular pathologies, orofacial pain, facial swelling, pain, nasal obstruction, and sinusitis [6]. In our instance, the patient had rhinosinusitis that was worsened by an anthrochoanal polyp. The ectopic tooth was discovered intraoperatively and during a radiological assessment after being missed during a routine inspection. Therefore, we should always keep odontogenic causes in mind and refer them to the oral maxillofacial team for further assessment. A symptomatic impacted ectopic tooth must be removed in order to avoid further issues and the recurrence of an underlying condition. However, the Caldwell-Luc procedure is the most common method utilised by surgeons to remove ectopic teeth from the maxillary sinus [6]. Other methods, such as nasal and transoral endoscopy, are available for removing ectopic teeth from the maxillary sinus. The complications of Caldwell Luc procedures are numerous and include facial swelling or numbness, facial & cheek discomfort, facial asymmetry, facial paraesthesia on the suborbital region, haemorrhage, oroantral fistula, dacryocystitis, and recurrence of rhinosinusitis due to the complex and constrained anatomy of the maxillary bone, which contains numerous significant neurovascular bundles.

Without a sufficient radiographic examination, it is impossible to make a diagnosis or schedule surgery; the current gold standard is still plain, conventional computed tomography films. Such imaging methods can be challenging to understand in certain circumstances, which is why 3D imaging has completely changed the way complex cases are treated. 3D printing and digital planning have several benefits. First, it serves diagnostic functions. The precise position and location of the ectopic tooth can be identified through 3D printing. In our example, correct anatomy and connections to the pterygomaxillary fissure could be detected due to their close proximity to nearby anatomical components. With this, surgeons could choose which surgical approach to use and whether their chosen surgical technique needs to be modified due to the anatomy. Additionally, the use of this 3D model during patient consent gathering and shared decision-making is possible. This enables us to provide an understandable presentation to the patient regarding the location of the ectopic molar tooth and to discuss in detail the various surgical options/approaches with the patient in order for them to decide [7]. A tangible help opportunity allows the patient to grasp concepts clearly.

 In our particular case, the patient also presented with rhinosinusitis complicated by anthrochonal polyp. In conjunction with the ectopic tooth extraction surgery, the patient required a functional endoscopic sinus surgery to address the sinus pathology; otherwise, the patient would present with disease recurrence. With the aid of 3D printing of the model, a multidisciplinary discussion could be held in a singular surgical setting to determine the optimal surgical approach. With this, we can reduce the time patients are exposed to general anaesthesia, thereby reducing their risk. In addition to 3D printing, this technology permits the modification of geometrical and anatomical details to optimise or facilitate preoperative planning for surgeons. With the availability of multiple programming applications, various implant properties can be optimised for a specific patient by modifying macro structures [8]. By modifying the image stereolithography processing in our case, we were able to remove the anthrochoanal polyp and precisely print only the aberrant molar tooth. This would allow the surgeon to determine the precise anatomical location of the ectopic molar tooth and assist in preoperative planning. We found that a patient's and doctor's perspective on a hand-held 3D model provided a greater understanding of the surgical situation than a software-reconstructed 3D model. Moreover, with an intervening antrochoanal polyp, it would be challenging to view and reproduce an image without an antrochoanal polyp, even with the bone window setting.

Multiple specialties have demonstrated that incorporating 3D models into pre-operative planning can enhance patient outcomes and facilitate communication between patients and surgical teams. Examples of the use of 3D modelling and printing in otorhinolaryngology include maxillary reconstruction, mandibular reconstruction, skull base surgery, temporal bone modelling, anterior skull base pathology modelling, and complex facial reconstruction [9]. Due to technological advancements, the turnaround time for these models is variable but still relatively fast, and they are now available in a variety of materials depending on their intended use. Using such models incurs additional costs; however, in our context, we have access to a 3D printing facility, and the approximate cost for a raw-cost printing a model of this size and material (PLA) is approximately 2USD, making it inexpensive and user-friendly.However, it's important to acknowledge that accessibility plays a significant role in leveraging this resource.

## Conclusions

This case report demonstrates a unique case of surgical planning using 3D printing in a case with a concomitant antrochoanal polyp and ectopic tooth. The 3D printing technology is ideally suited for locating and visualising the ectopic molar tooth. Digital planning and 3D printing are beneficial because they allow for diagnosis, preoperative treatment planning, and use in the consent process. This could lead to successful surgeries with minimal risk, resulting in an enhanced patient outcome.
